# T2 mapping of molecular subtypes of WHO grade II/III gliomas

**DOI:** 10.1186/s12883-019-1590-1

**Published:** 2020-01-08

**Authors:** Maike Kern, Timo Alexander Auer, Thomas Picht, Martin Misch, Edzard Wiener

**Affiliations:** 1 Department of Neuroradiology, Charité – Universitätsmedizin Berlin, corporate member of Freie Universität Berlin, Humboldt-Universität zu Berlin and Berlin Institute of Health, Berlin, Germany; 2 Department of Radiology, Charité – Universitätsmedizin Berlin, corporate member of Freie Universität Berlin, Humboldt-Universität zu Berlin and Berlin Institute of Health, Berlin, Germany; 3 Department of Neurosurgery, Charité – Universitätsmedizin Berlin, corporate member of Freie Universität Berlin, Humboldt-Universität zu Berlin and Berlin Institute of Health, Berlin, Germany

**Keywords:** Gliomas, MRI, IDH, T2-mapping

## Abstract

**Background:**

According to the new WHO classification from 2016, molecular profiles have shown to provide reliable information about prognosis and treatment response. The purpose of our study is to evaluate the diagnostic potential of non-invasive quantitative T2 mapping in the detection of IDH1/2 mutation status in grade II-III gliomas.

**Methods:**

Retrospective evaluation of MR examinations in 30 patients with histopathological proven WHO-grade II (*n* = 9) and III (*n* = 21) astrocytomas (18 IDH-mutated, 12 IDH-wildtype). Consensus annotation by two observers by use of ROI’s in quantitative T2-mapping sequences were performed in all patients. T2 relaxation times were measured pixelwise.

**Results:**

A significant difference (*p* = 0,0037) between the central region of IDH-mutated tumors (356,83 ± 114,97 ms) and the IDH-wildtype (199,92 ± 53,13 ms) was found. Furthermore, relaxation times between the central region (322,62 ± 127,41 ms) and the peripheral region (211,1 ± 74,16 ms) of WHO grade II and III astrocytomas differed significantly (*p* = 0,0021). The central regions relaxation time of WHO-grade II (227,44 ± 80,09 ms) and III gliomas (322,62 ± 127,41 ms) did not differ significantly (p = 0,2276). The difference between the smallest and the largest T2 value (so called “range”) is significantly larger (p = 0,0017) in IDH-mutated tumors (230,89 ± 121,11 ms) than in the IDH-wildtype (96,33 ± 101,46 ms). Interobserver variability showed no significant differences.

**Conclusions:**

Quantitative evaluation of T2-mapping relaxation times shows significant differences regarding the IDH-status in WHO grade II and III gliomas adding important information regarding the new 2016 World Health Organization (WHO) Classification of tumors of the central nervous system. This to our knowledge is the first study regarding T2 mapping and the IDH1/2 status shows that the mutational status seems to be more important for the appearance on T2 images than the WHO grade.

## Background

Malignant gliomas are the most frequent primary brain tumors in adults [1–6]. In 2016 the World Health Organization (WHO) Classification of Tumors of the Central Nervous System initially “integrated” [7] genotypic parameters such as molecular genetic tumor markers in its revised version from 2016 [5, 8, 9]. This new classification adds the genetic markers and gives a more precise division into molecular subgroups with distinct molecular signatures [5, 8, 10]. These groups may correlate with their histologic subtype and define different patient’s prognosis and treatment response [5, 8–12].

One of the most important markers in gliomas is the isocitrate dehydrogenase (IDH) 1 and 2 [5, 9, 11–13]. IDH1 and IDH2 are coding for isocitrate dehydrogenase, which catalyzes the conversion of isocitrate to alpha-ketoglutarate (ΑKG), leading to an increased level of D2HG in IDH mutated tumors [3, 5, 6, 13–15]. Another effect of IDH-mutation (IDH-mut) is the inhibition of the PI3K/Akt pathway, which may induce a higher level of apoptosis [16]. IDH mutations in general are associated with a better prognosis by influencing cell proliferation, angiogenesis and vascularization [1, 3, 5, 6, 8, 9, 11, 14, 16–18].

High-grade gliomas treatment response used to be evaluated with the Macdonald-Criteria from 1990 [4, 19, 20]. In 2010, the Response Assessment in Neuro-Oncology (RANO) working group published the updated RANO criteria, that replaced the Macdonald-Criteria [4, 19–21]. RANO criteria include the T2/FLAIR (fluid-attenuated inversion recovery) sequences in addition to the recent clinical and imaging features [4, 19, 20]. Until then, imaging features used just T1w and T1w contrast-enhanced sequences [4, 19, 20]. The RANO- criteria now include a significantly increased T2/FLAIR perpendicular size (called “T2-progress”) as a criterion [4, 19, 20]. As a future aspect T2 mapping could affect the evaluation of tumor progression according to RANO criteria by adding quantitative voxelvise measurements. Nevertheless, both classification systems are constructed to correlate with the histological grade and not with a genetic profile.

The importance of T2 mapping sequences was already emphasized by Hattingen et al. (2013) [22]. Their study shows, that this technic could be suitable to control the tumor progression under anti-angiogenetic therapy [22]. According to their results, by use of T2 maps it seems achievable to detect tumor progression by an increase in T2 relaxation times in healthy appearing brain tissue before demarcated changes are visible in usual MRI sequences [22]. Further examinations about the possible applications of this promising technic, especially in neuroradiological usage, are urgently needed.

The future key role in MR brain imaging will be to create imaging biomarkers for defining correct molecular subtypes.

The purpose of this study is to evaluate the potential of quantitative T2-mapping in the detection of IDH1/2 mutation status in grade II-III gliomas. In the new WHO classification, molecular profiles are providing reliable information about prognosis and treatment response. To the best of our knowledge, it is the first study for initial results by use of T2 mapping to demonstrate, that the mutational status causes characteristic alterations in the T2 values of glioma.

## Methods

### Study population

**Fig. 1:**
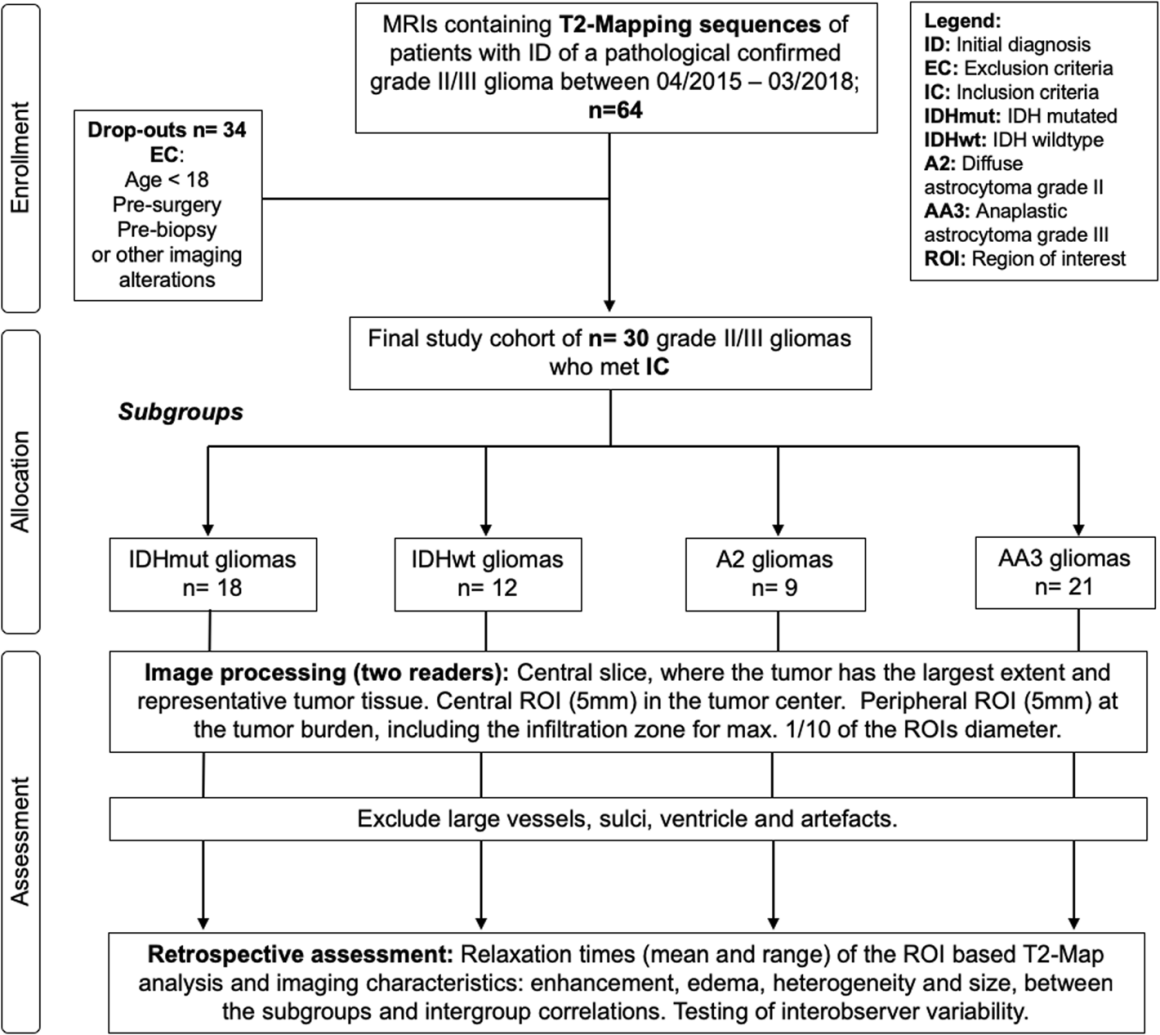
Flowchart of the process of data enrollment, allocation, and assessment

The ethical board of our institution approved the present study (application number EA1/306/16). All participants were enrolled after their informed consent was obtained. The study was conducted according to the Declaration of Helsinki in its revised revision. Patients with the diagnosis of WHO grade II astrocytoma (A2) and WHO grade III anaplastic astrocytoma (AA3) were identified within the period from April 2015 to March 2018 (Fig. 1). A total of 22 patients with A2 and 42 patients with AA3 were enrolled. Patients younger than eighteen years of age, with a previous central nervous surgical treatment with imaging alterations such as artefacts, considerable surgical cavity or mass effect were excluded. In total 9 patients with A2 and 21 patients with AA3, including 18 with and 12 without IDH mutation, were included. Histopathological reports including molecular typing were available for all patients.

### Data acquisition

MR imaging was performed preoperatively on 1.5 Tesla (Avanto Magnetom; Siemens, Erlangen, Germany) (*n* = 20) and 3 Tesla (Skyra; Siemens, Erlangen, Germany) (*n* = 10) scanners with and without contrast media. Dotarem (Guerbet GmbH) or Gadovist (Bayer Healthcare) were administered weight adapted. Following sequences were acquired: T1 magnetization-prepared rapid gradient echo (MPRAGE) transversal, sagittal and coronar post contrast (repetition time (TR) 2200, echo time (TE) 2,67, slice thickness 1 mm, inversion time 900, in-plane resolution 0,9766 × 0,9766 mm, acquisition matrix 256 × 246) and T2 mapping (TR 3100 TE 13,8–165,6 with twelve TEs: 13,8 ms, 27,6 ms, 41,4 ms, 55,2 ms, 69 ms, 82,2 ms, 96,6 ms, 110,4 ms, 124,2 ms, 138 ms, 151,8 ms, 165,6 ms). T2 maps were reconstructed online by using a voxelwise, monoexponential nonnegative least-squares fit analysis (MapIt; Siemens, Erlangen, Germany) with a voxel size of 1.9 × 1.0 × 3 mm^3^.

### Image analysis

**Fig. 2:**
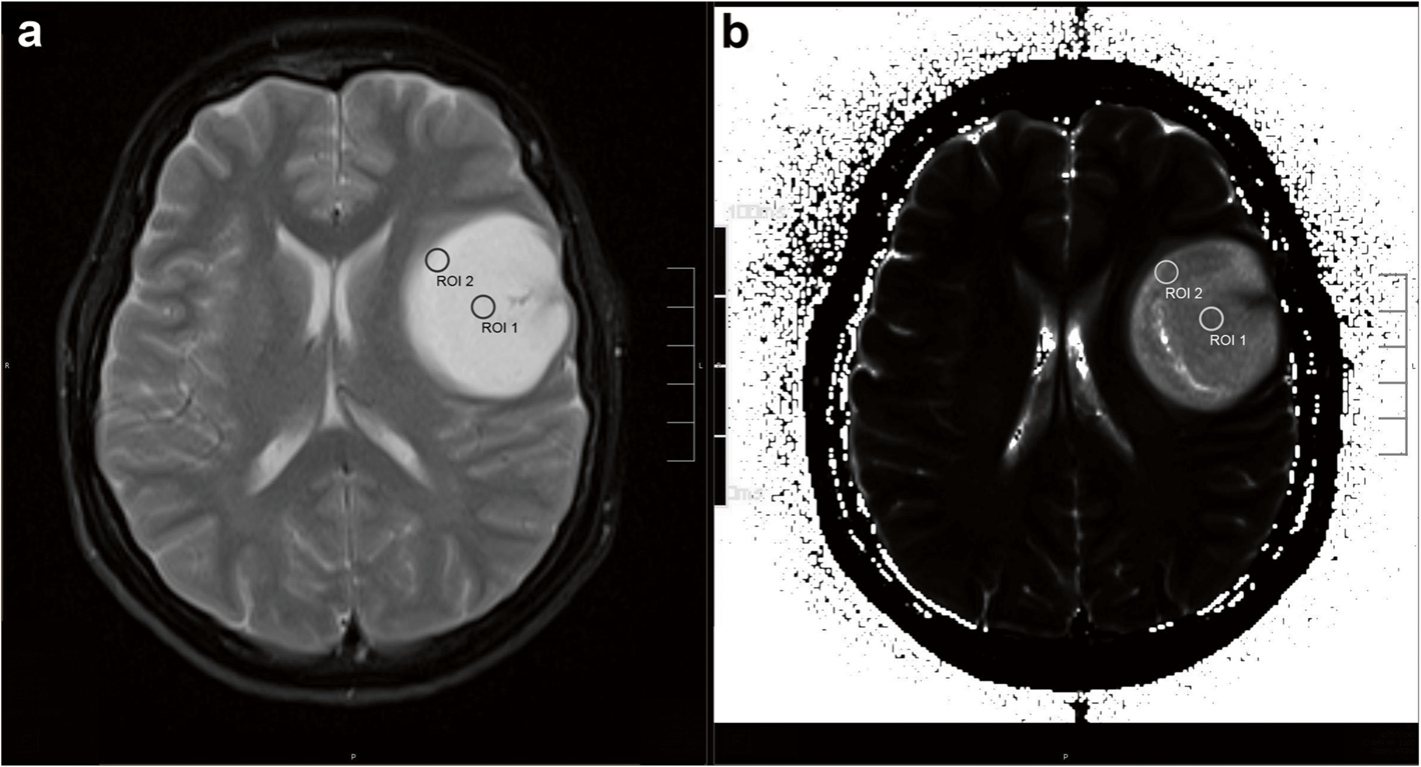
MRI of a 32 years old female patient suffering from IDH-mut anaplastic astrocytoma WHO grade III. The T2 hyperintense tumor shows no enhancement or necrosis and only small edema can be assumed. The central ROI and peripheral ROI are delineated on the transversal T2 image (**a**) and on the corresponding T2 map (**b**)

For quantitative analysis, regions of interest (ROI) were manually drawn (M.K., E.W.) on the T2-maps using the visage software tool (Visage Imaging/Pro Medicus Limited, Version 7.1.10). To test the accuracy of the T2 measurements, an additional ROI was placed in the healthy white matter (WM) of the contralateral lobe on a level without the ventricles. In the manner of Badve et al., we preferred the contralateral equal lobe [23]**.** If there was not any normal appearing white matter to be found, we placed the ROI with a different localization, most often frontoparietal [23]**.** To proof the reliability of our T2 measurements, we took the median value for the WM which supports recent studies which reported ranges from 84 to 87 ms ± 3 ms in 1.5 T MRI [24] and 74 to 80 ms ± 1 ms in 3.0 T [25]. Additionally, we compared the WM values of 1,5 T and 3 T MRI to rule out the source of error occurring through the mixture of field strengths. Next, two different locations for the ROI’s were selected, a central ROI (cROI) and a peripheral ROI (pROI) based on the following criteria (Fig. 1). The ROI size was always 5 mm in diameter. For the cROI, the slice with the largest tumor dimension was chosen, followed by delineating of the anatomic center of the tumor. In case of present vessels, necrosis or artefacts, the cROI was placed a few millimeters off center. The pROI was placed on the same slice as the cROI. The pROI was located within the outer tumor border including the infiltration zone with 1/10 of the ROI, always bright on T2 maps and directly adjacent to healthy appearing brain tissue (Fig. 1 and Fig. 2). Vessels, artefacts and sulci were excluded as before (Fig. 1). All ROI’s covered parts that were representative of the whole tumor’s tissue. IDH mutation was defined by the use of immunohistochemistry. If the result was obscure, it was followed by a PCR and pyrosequencing analysis. Furthermore, enhancing tumor (yes/no), necrosis (yes/no), edema (yes/no), tumors heterogeneity (on a scale from low (1) to high (3)) and tumor size on the MPRAGE images were evaluated. We defined a signal heterogeneity of more than 25% (visually) in either T1w or T2w/FLAIR sequences as a macroscopic heterogenic pattern [1, 26, 27]. Both observers were constantly blinded to IDH status and the diagnosis during the measurements.

### Statistical analysis

The statistical analysis was performed with XLSTAT (Version 2011,0,01; Addinsoft SARL, New York, New York). The Mann-Whitney-U Test as a two-tailed test was used to compare each groups median. Kruskal-Wallis-Test was used for variance analysis/ rank sum test. *P* < 0,05 was evaluated as statistical significance. Interobserver variability was calculated using Cronbach’s alpha statistic with the values for parenchyma, central and peripheral ROI. Cohen’s d was used to calculate the effect size of the significant parameters on the T2 values of gliomas. The analysis of variance (ANOVA) was used to analyze differences among the group means.

## Results

### Study subjects

General information of our study subjects for the separate tumor entities is summarized in Table 1. Overall there are more patients with IDH-mut (*n* = 18) than with IDH-wt (*n* = 12). Frontal and parietal (*n* = 12) localization prevail significantly in IDH-mut tumors (*p* < 0,0001). The patients with IDH-mut (41,32 ± 12,3 [28–74] years) were significantly younger than those with IDH-wt (55,69 ± 16,39 [34–81] years) (*p* = 0,0105).

**Table 1:** General data of the study population

	IDH-mut (*n* = 18)	IDH-wt (*n* = 12)	*p*-value*
age, yr. (mean, range)	41,32 ± 12,3 [28–74]	55,69 ± 16,39 [34–81]	0,0105
A2	38,13 ± 11,53 [28–57]	64,22 ± 18,25 [41–81]	
AA3	42,55 ± 12,81 [29–74]	51,42 ± 14,73 [34–74]	
sex (no.)	*n* = 18	*n* = 12	< 0,0001
female	9 (50%)	1 (8,33%)	
male	9 (50%)	11 (91,67%)	
tumor grade	*n* = 18	*n* = 12	
A2	5 (27,78%)	4 (33,33%)	
AA3	13 (72,22%)	8 (66,67%)	
tumor localization	*n* = 18	*n* = 12	< 0,0001**
fronto-parietal	frontal (38,89%) parietal (16,67%) frontoparietal (11,1%)	frontal (16,67%) parietal (16,67%)	
other	fronto-parieto-temporal (16,67%) temporal (11,1%) parieto-temporal (5,56%)	thalamic (25%) temporal (16,67%) frontotemporal (8,33%) corpus callosum (8,33%) 5 single lesions (8,33%)	

*All *p*-values relate to the difference between IDH-mut and IDH-wt independently of the tumor grade. **This *p*-value concerns to fronto-parietal or not fronto-parietal localization in IDH-mut and IDH-wt tumors

### MR examination

**Fig. 3:**
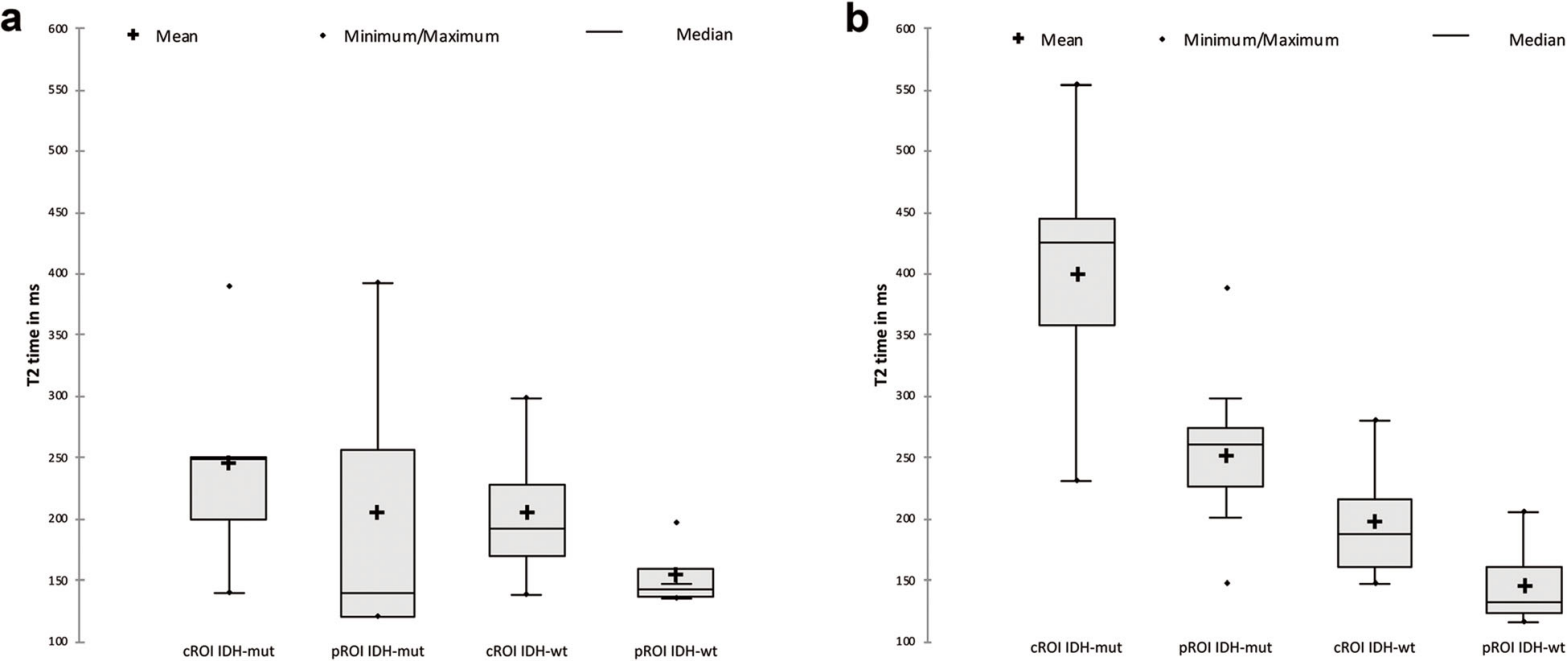
Box plots of T2 values of IDH-mut and IDH-wt obtained from central ROIs and peripheral ROIs of a WHO grade II astrocytomas (**a**) and WHO grade III astrocytomas (**b**)

**Fig. 4:**
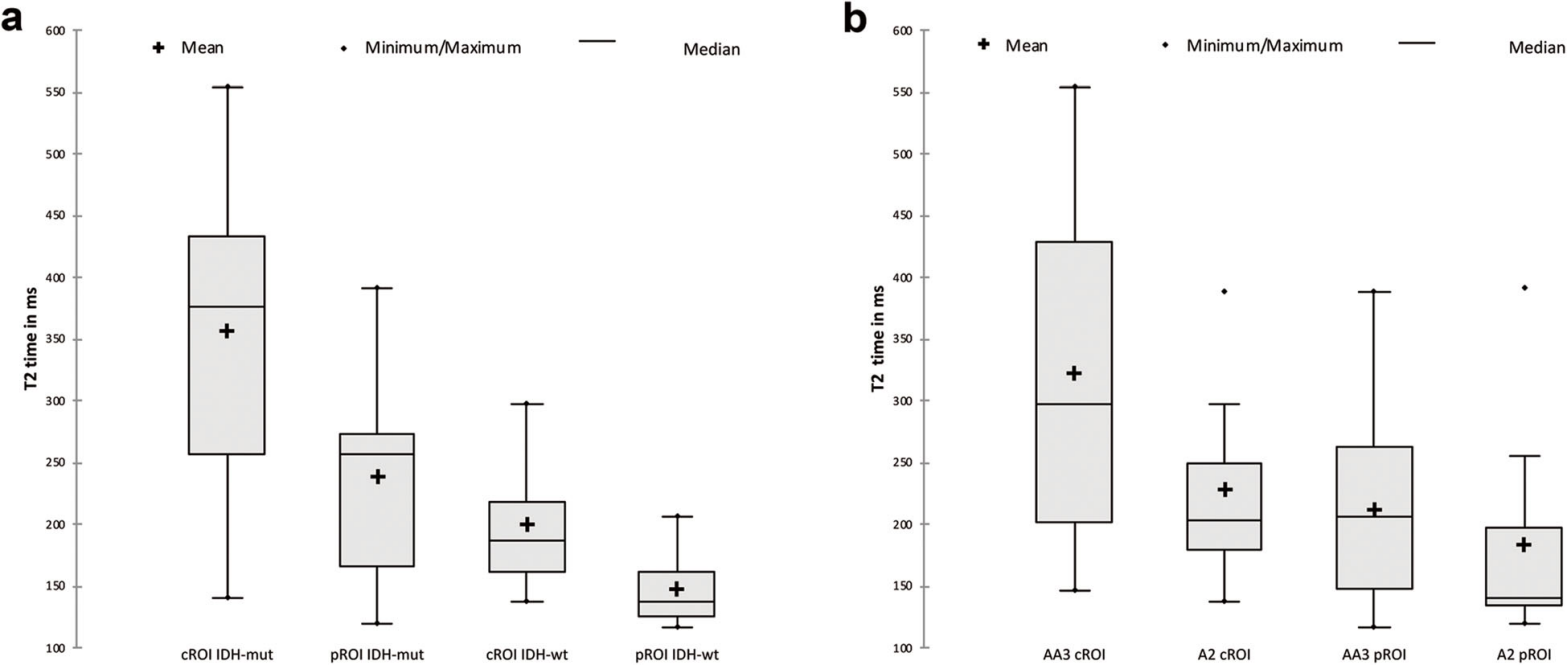
Box plots of T2 values obtained from central ROIs and peripheral ROIs of IDH-mut and IDH-wt without the distinction between WHO grade II and III tumors (**a**) and analysis of T2 values only considering the WHO grade without the IDH status (**b**)

Figure 2 shows a T2 weighted image and the corresponding T2 map of a WHO grade III astrocytoma with IDH mutation. The median T2 values differed significantly between the central and peripheral ROI in gliomas of both grades (*p* = 0,0021), IDH-mut (*p* = 0,0288) and IDH wt (*p* = 0,0362) and in AA3 (p = 0,0185). In A2, there was no significant difference even if the figures show similar trends (*p* = 0,4089) (Table 2, Fig. 3 and Fig. 4). The T2 values were larger in the center ROI than in the peripheral ROI. This difference represents the SI decreasing from the center to the periphery, which occurs independently of the IDH-status (Table 2). Separated by the mutational status, the IDH-mut gliomas are central (*p* = 0,0037) and peripheral (*p* = 0,0094) more intense than the IDH-wt (Table 2, Fig. 3 and Fig. 4). IDH-mut gliomas are overall significantly more hyperintense than the wildtype (Table 2, Fig. 3 and Fig. 4). There was no significant difference between A2 and AA3, neither central (*p* = 0,2276) nor peripheral (*p* = 0,5547) (Fig. 3). Fig. 3 shows the previously mentioned trends in A2 (central vs. peripheral *p* = 0,4089, central mut vs. wt *p* = 0,8831; peripheral mut vs. wt *p* = 1,0000).

**Table 2:** T2 values of ROIs from different location and tumor entities in dependence of the molecular subtype and the calculated *p*-values

	mean [median] T2 ± StD	mean [median] T2 ± StD	*p*-value
central vs. peripheral ROI			
both grades	294,07 [268,50] ± 122,2 ms	202,6 [186,50] ± 78,71 ms	**0,0021**
IDH-mut	356,83 [376,50] ± 114,97 ms	239,00 [257,00] ± 80,44 ms	0,0288
IDH-wt	199,92 [187,50] ± 53,13 ms	148,00 [138,00] ± 30,22 ms	0,0362
AA3	322,62 [298,00] ± 127,41 ms	211,10 [206,00] ± 74,16 ms	0,0185
A2	227,44 [204,00] ± 80,09 ms	182,78 [140,00] ± 89,87 ms	0,4089
IDH-mut vs. IDH-wt			
central ROI	356,82 [376,50] ± 114,97 ms	199,92 [187,50] ± 53,13 ms	**0,0037**
peripheral ROI	239,00 [257,00] ± 80,44 ms	148,00 [138,00] ± 30,22 ms	0,0094
AA3 central ROI	399,69 [425,00] ± 93,46 ms	197,38 [187,50] ± 49,47 ms	0,0022
AA3 peripheral ROI	251,85 [261,00] ± 61,93 ms	144,88 [132,00] ± 32,28 ms	0,0061
A2 central ROI	245,40 [249,00] ± 92,08 ms	205,00 [192,00] ± 67,74 ms	0,8831
A2 peripheral ROI	205,60 [140,00] ± 118,58 ms	154,25 [142,5,00] ± 28,95 ms	1,0000
range IDH-mut vs. IDH-wt			
central both grades	230,89 [249,50] ± 121,11 ms	96,33 [56,50] ± 101,46 ms	**0,0017**
central AA3	262,23 [273,00] ± 111,69 ms	69,63 [49,50] ± 43,39 ms	0,0042
central A2	149,40 [102,00] ± 116,80 ms	149,75 [79,50] ± 166,28 ms	0,9614

Bold data shows significant values

The range of the measurements is larger in IDH-mut than it is in IDH-wt (*p* = 0,0017; *p* = 0,0042) (Table 2, Fig. 3 and Fig. 4).

The median T2 value of white matter has been 82,8 ± 3,13 [78–89] ms. There was no significant difference between the T2 values of WM obtained at 1,5 T (83,6 ± 3,1 [80–89] ms) and 3 T (81,3 ± 2,4 [78–86] ms) MRI (*p* = 0,053).

We had good interobserver agreement by comparison of T2 values of parenchyma, central and peripheral ROI (α = 0,9509).

There was a significant difference in the enhancement, IDH-wt gliomas (*n* = 4) had more CE than IDH-mut (*n* = 4) (*p* < 0,0001). However, there was significantly more necrosis in IDH-mut than in IDH-wt astrocytomas (*p* < 0,0001). There was no significant difference in macroscopic heterogeneity between A2 (1667 ± 0,866) and AA3 (1857 ± 0,793) (*p* = 0,583) nor between IDH-mut (1,78 ± 0,88) and IDH-wt (1,83 ± 0,72) (*p* = 0,7957) based on a nominal scale.

In this study the calculated Cohen’s d of 1.506 and 1.766 represent a large effect size, indicating a strong impact of IDH status on T2 values of gliomas. ANOVA revealed no statistically significant impact of age (*p* = 0.5730), gender (*p* = 0,8461) and localization (*p* = 0,6145) on the T2 values of gliomas. The WHO grade revealed a small impact on T2 values in ANOVA variance analysis (*p* = 0,472).

## Discussion

To our knowledge this is the first study, that quantitatively compared the diagnostic utility of T2 mapping sequences in distinguishing between WHO grade II and III astrocytomas in relation to the IDH status demonstrating a significant T2 increase in IDH-mut gliomas compared to the wild type. Furthermore, a significant difference between the central and peripheral tumor regions was found. Although, histopathological tissue examination remains the diagnostic gold standard, T2 mapping is a noninvasive examination and provides additional information about the macroscopic and microscopic composition [28, 29].

The new WHO classification from 2016 emphasizes the prognostic value of molecular subtypes. Current literature states that the IDH mutation is associated with a better prognosis and therapeutic response [5, 8, 14].

It is reported that patients with IDH-mut AA3 had a longer survival than with IDH-wt AA3 [14]. Several studies [11, 14, 29, 30] showed that the mutation is associated with younger age though (*p* = 0,02), which is in line with our results.

MRI is the modality of choice in the clinical diagnosis, treatment planning, and follow-up of brain tumors. Sufficient differentiation of grade II and III gliomas using conventional MRI remains challenging, due to similar imaging presentations as both grades are able to enhance and both grades may grow necrotic [28]. In agreement with the study of Qi et al. (2014) only for necrosis and CE could be found significant correlations, whereas edema and tumor size did not show significant results [1, 5].

The data show that A2 have lower intensities than AA3 but without significant results. Further studies who investigated the T2-volume didn’t show convincing results [31]. Patel et al. (2018) did not find differences in the segmented volume of T2 hyperintensity in relation to the WHO grade [31]. Therefore, it seems that there is no evidence for the differentiation between WHO grade II and III gliomas based on T2 signal characteristics.

Earlier reports suggested highly promising clinical utility for MR spectroscopy and Positron Emission Tomography (PET)-CT [3, 5, 30, 32, 33]. Verger et al. (2017) for example showed an increased Tracer (F-FDOPA) uptake in PET-CT in IDH-mut and WHO grade III glioma [30]. Even recent studies like Villanueva-Meyer et al. (2018) who found significant differences depending on the IDH status of WHO grade II glioma by use of ADC (apparent diffusion coefficient)-maps could not fully meet the expectations in clinical practice [9]. Nevertheless, no technique was valid enough to implement it into the clinical routine.

Our T2-map analyses show that IDH-mut AA3 have the highest SI, followed by IDH-mut A2, which are similar to IDH-wt AA3. IDH-wt A2 yield the lowest values. The median intensities of IDH-mut A2 are nearly equal to the ones of IDH-wt AA3. Furthermore, the comparison of the values of the cROI of A2 vs. AA3 (*p* = 0,2276) and IDH-mut vs. IDH-wt (*p* = 0,0037), demonstrates as well as the ANOVA analysis, that the mutational status seems to be more important for the SI than the WHO grade, which is surprising. The WHO grade describes different stages within the progression of the same condition, emphasizing the mutational status.

The IDH mutation might be accountable for several changes in tumors cell biology and metabolism. As several previous studies mentioned, we presume that the evident T2 signal characteristic of IDH-mut tumors and tumor cells especially in the central region is caused by the accumulation of D2HG and the changed tumor metabolism [30, 34]. Increased vascularization, angiogenesis and apoptosis may cause edema, which is T2 hyperintense, too. Nevertheless, there are lower values in the peripheral tumor region. That might implicate, that there is a mixture of parenchyma with a lower SI and tumor cells with a higher SI leading to the hypothesis that gliomas always grow infiltrative from the inside out [11]. Parenchyma cells depress the overall values. We guess that the larger range of IDH-mut AA3 and IDH-mut tumors in general, could express a microscopic heterogeneity. This IDH-specific heterogeneity was also postulated by Darlix et al. (2017), caused by different factors [12]. First, throughout the inhibited PI3K/Akt pathway resulting in a higher rate of apoptosis, so there might be macroscopically invisible necrosis, which could be presented in lower T2 values [16]. Secondly, the stabilized HIF-1α increases vascularization and angiogenesis and may also result in heterogeneity [3, 14, 17]. Interestingly, the macroscopic heterogeneity did not vary in our cohort from IDH-mut to IDH-wt nor between A2 and AA3. Heterogeneity is used as another radiological criterion to differ between the WHO grades but might be marginal in differentiation between WHO grade II and III or IDH-mut and IDH-wt glioma. Although Wang et al. (2015) reported, the IDH-wt tumors are mostly located multilobar, frontal and temporal [11, 35]. In this study, the IDH-mut tumors are significantly more often located frontal and parietal region (Table 1), in line with the current literature [29, 35].

By use of two different locations for ROI analysis, we can show that it does not matter where the ROI-analysis inside the tumor area is performed. The RANO criteria firstly include the T2/FLAIR, showing that T2 weighted images are just as important as the T1 weighted postcontrast. It seems as T2 signal characteristics are at least as important as the tumor size.

Limitations of the study must be acknowledged. Our study sample included a minor portion of patients who were operated years ago with no considerable surgical cavity or mass effect visible in the included brain MRI. Further potential sources of bias in our study are selection bias, detection bias and lack of previous research studies on the topic. In addition, there is uneven sex distribution. Another limitation is the small sample size of patients, even if the effect size is already large. The remaining goldstandard, the histopathological classification, is known to have high inter- and intraobserver concordances [29]. The small interobserver variability in this study shows, that the technique of T2 mapping is reliable, reproducible and not observer-depending. The visual based and not volumetric evaluation is another possible bias. The comparison of the values of brains parenchyma between 3 T and 1,5 T scanners shows, that there is no significant difference among the technics regarding T2 mapping and that T2 mapping is a very robust technique for clinical routine. T2 values of brain parenchyma may not be affected so much by the field strength [36] . Due to the performance with two different scanners, it is possible that they had slightly different pulse sequences and settings, which might influence the results as well.

Like Villanueva-Meyer et al. (2018) and Qi et al. (2014) reported, newer imaging techniques, localization, growth behavior, margins, and CE are also predictive for the IDH-status [1, 5, 9, 11, 35]. Our results obtained out of mapping data might add important knowledge at this actual topic.

## Conclusion

Quantitative evaluation of T2-mapping relaxation times shows significant differences regarding the IDH-status in WHO grade II and III gliomas as IDH-mut gliomas have a higher T2 SI, adding important information regarding the new 2016 World Health Organization (WHO) Classification of tumors of the central nervous system. Furthermore, T2 mapping seems to quantify the heterogeneity of grade III gliomas. This to our knowledge first study regarding T2 mapping and the IDH1/2 status shows that the mutational status seems to be more important for the appearance on T2 images than the WHO grade.

## Data Availability

The data that support the findings of this study are available on request from the corresponding author MK.

## Abbreviations

A2: WHO grade II diffuse astrocytoma
AA3: WHO grade III anaplastic astrocytoma
CE: Contrast enhancement
cROI: Central region of interest
CT: Computer tomography
D2HG: D-2-hydroxyglutarate
FLAIR: Fluid-attenuated inversion recovery
HIF- 1Α: Hypoxia-inducible factor − 1 alpha
IDH: Isocitrate dehydrogenase
IDH-mut: Mutational IDH
IDH-wt: IDH-wildtype
MPRAGE: Magnetization-prepared rapid gradient echo
MRI: Magnet resonance imaging
PCR: Polymerase chain reaction
pROI: Peripheral region of interest
RANO: Response Assessment in Neuro-Oncology
ROI: Region of interest
SI: Signal intensity
StD: Standard deviation
T: Tesla
TE: Echo time
TR: Repetition time
WHO: World Health Organization
WM: White matter
ΑKG: Alpha-ketoglutarate
